# It’s what you do, not the way you do it – online versus face-to-face small group teaching in first year medical school

**DOI:** 10.1186/s12909-021-02981-5

**Published:** 2021-10-26

**Authors:** Adrienne Torda, Boaz Shulruf

**Affiliations:** Faculty of Medicine and Health, UNSW Sydney, Randwick, Australia

**Keywords:** Medical education, Online learning, Educational design, Engagement, Wellbeing, Social outcomes

## Abstract

**Background:**

Major disruptions imposed on medical education by the COVID-19 pandemic and the rapid shift to online teaching in medical programs, necessitated need for evaluation of this format. In this study we directly compared knowledge outcomes, social outcomes, and wellbeing of first year student small group teaching in either face to face (f2f) or online format.

**Methods:**

At the end of the first course of our medical program, students were invited to participate in an online questionnaire with 10 quantitative items and 1 qualitative item. These were analysed using Factor Analysis Pattern Matrix and linear regression to group items and assess relatedness. Qualitative responses were thematized using Qualtrics software (Qualtrics, Provo, UT, USA). Summative assessment results were compared, both between current cohorts to historical cohorts.

**Results:**

From a cohort of 298 students there was a 77% response rate. Overall, there were no differences in knowledge gains, either between groups or when compared to historical cohorts. Questionnaire items fell reliably into groups that related to either learning outcomes, social outcomes, or wellbeing. Independent T tests showed that format for teaching (online versus f2f) had an impact on social outcomes but no direct impact on learning outcomes. Linear regression revealed that the social outcomes have a direct impact on wellbeing and almost the double the impact on learning outcomes than mode of learning i.e.. F2f or online (β = .448 and β = .232 respectively).

**Conclusion:**

In this study, we were able to show with statistical strength that social outcomes for students such as engaging with peers and facilitator, contributing to the group, and making friends have a direct impact on wellbeing and indirectly impact learning outcomes (such as motivation, satisfaction, integration of knowledge). In a rapidly changing educational landscape, in our opinion, it is vital that these aspects are a focus of design and delivery of medical education. The data from this study supports the notion that activity design and the expertise of the teacher in facilitating the small group activities, has greater impact than the mode of educational delivery itself on students’ learning processes.

**Supplementary Information:**

The online version contains supplementary material available at 10.1186/s12909-021-02981-5.

## Background

Like many other medical faculties, during the COVID-19 pandemic, campus-based courses in the junior years of our medical program were transitioned online. Prior to the COVID-19 pandemic, very little of our medical program was online, even for the junior, primarily campus-based years. There were some self-directed, interactive, online learning activities built into practical classes and clinical disciplines, but other than that, learning activities were essentially delivered in face to face (f2f) mode. This all changed in 2020 in response to campus rapidly closing, then, as our campus re-opened, these changes were reviewed and the cornerstone learning unit of junior years of our medical program, our scenario learning groups [1], were re-established as face to face activities for all students, except those that were still offshore due to ongoing international travel restrictions. It is likely that more disruptions will occur throughout the ebbs and flows of this pandemic. To minimise disruption of medical education, educators will need to continue to build resilience into the way they deliver medical education, evaluate, and produce data around contemporary ‘best practice’ and how this looks in a pandemic-constrained environment [2].

During the first 2 years of our (undergraduate) medical program, the courses are all inter-disciplinary and contain a variety of learning activities including lectures, practical classes, clinical and communication skills sessions, and scenario-group sessions (SGs). The SGs have a variety of names such as problem-based learning. This approach is commonly used in medical programs [3]. SGs are considered to be key learning sessions in medical training programs for many reasons, largely because the students are developing skills in small groups for self-directed and lifelong learning, which include problem solving, critical thinking, team-based learning and reflection, as well as delivery of content [4, 5]. The SGs are essentially designed to enable students to learn and revise course content and develop learning skills, using active learning principles such as self-directed learning, problem solving, teamwork and reflection, under the direction of a facilitator. This session is thought to be the most important in the junior years of the course, both for the development of skills such as these learning skills and also for the establishment of relationships with other students, friendships and relationships with teachers [6, 7]. It is during these SG sessions that students generally have the most opportunity to develop learning skills and also for collaborative learning, sharing knowledge and what can be called communal ‘constructivism’ in relation to learning [8]. For this reason, we undertook this study to assess the impact of these SGs being taught online as opposed to face to face (f2f).

In 2021, almost one third of our first-year student cohort were still offshore, unable to physically enter Australia due to international travel restrictions. All educational activities in the first course of the medical program were still being conducted online at that time, apart from the SGs. This allowed us to directly compare impact of the two formats of this key learning activity. We conducted a study to examine the impact of the mode of delivery (online versus f2f) on learning outcomes, learning outcomes, social outcomes, and wellbeing.

## Methods

At the end of the Foundations course in the undergraduate medical program, all enrolled students (298) were invited to participate in a web-based survey (Additional file 1: Appendix A) which was created using Qualtrics software (Qualtrics, Provo, Utah, United States) and distributed to all students via internal notification in April 2021. The survey featured Likert scale questions about students’ experience of learning outcomes, social engagement, and wellbeing. This survey was adapted from the Perceived Utility of Learning Technologies Scale (PULTS) which was previously developed and validated within the UNSW Faculty of Medicine’s Blended Learning Project. The PULTS survey assesses perceptions of and engagement with learning resources to gather student feedback. There were 10 quantitative items in total, which could be grouped into categories of either learning outcomes or social outcomes and one on wellbeing (summarised in Table 1). Qualitative data was also collected about the best features of these learning sessions. The responses were then filtered according to whether the student was in a f2f or online group and responses of the two cohorts were compared. Objective knowledge gains of students were also assessed by summative end of course assessments were compared between the two current cohorts and compared to historical cohorts 2019 and 2020).

**Table 1:** Factor Analysis Pattern Matrix^a^: Student perception of their learning experiences

Questionnaire item	Factor	
	1	2
**Q4 Activities Enhanced Learning**	**.97**	−.13
**Q5 Useful for Integrating Knowledge**	**.92**	−.10
**Q3 Enhanced Motivation to Learn**	**.75**	.14
**Q1 Satisfied with SG Sessions**	**.63**	.28
Q10 Enhanced Wellbeing	.48	.43
*Q9 Easy to Make Friends*	−.22	***.93***
*Q8 Easy to engage with other students*	−.06	***.90***
*Q6 Easy to contribute to Group*	.12	***.59***
*Q7 Easy to Engage with Facilitator*	.14	***.58***
Q2 Enjoyed Learning Format	.24	.43
Reliability Cronbach’s alpha	**.91**	***.85***

Extraction Method: Maximum Likelihood. Rotation Method: Oblimin with Kaiser Normalization. a. Rotation converged in 6 iterations

### Statistical analysis

Exploratory Factor analysis (Maximum Likelihood, using Oblimin Rotation with Kaiser Normalization) was employed to identify the underlying factors of student perceptions. Independent t-tests were used to measure the differences in student perceptions between the f2f and online groups. A multiple linear regression model was used to identify the predictors of learning outcomes, and path analysis, using structural equation modelling, was used to identify direct and indirect impacts on student wellbeing. Analyses were conducted by SPSS v22 and R [9–11].

## Results

From a cohort of 298 students there were 213 respondents (77% response rate) with a total completion rate of 97.7%. Of participants that completed the survey, 143 were f2f and 65 were online which represented very good and equitable sampling across both groups. The raw results of the Likert questions are shown in Additional file 2: Appendix B.

### Impact of f2f versus online learning on learning and social outcomes

An exploratory factor analysis was done to assess the contribution of the factors to learning outcomes and social outcomes (Table 1).

This table shows the significant groupings of questionnaire items into those that relate to the learning outcomes (bold) and those that relate to the social outcomes (italic). We were able to use these groups for factor analysis and assess their impact on each other. The reliability of each factor is high, apart from items 2 and 10 in this analysis.

An independent T test was performed to see if the format for teaching (online versus f2f) had an impact on these outcomes. They show a significant impact on the social outcomes but not the learning outcomes as shown in Table 2.

**Table 2:** Independent Samples Test: Impact of teaching format on learning and social outcomes

		Levene’s Test for Equality of Variances		t-test for Equality of Means						
		F	Sig.	t	df	Sig. (2-tailed)	Mean Difference	Std. Error Difference	95% Confidence Interval of the Difference	
									Lower	Upper
Learning Outcomes	Equal variances assumed	2.00	.16	−1.63	206	**.115**	**−.23**	.14	−.50	.05
	Equal variances not assumed			−1.81	160.74	.07	−.23	.13	−.47	.02
Social Outcomes	Equal variances assumed	19.31	.00	3.97	206	.00	.48	.12	.24	.72
	Equal variances not assumed			3.28	84.11	**.00**	**.48**	.15	.19	.77

**Fig. 1:**
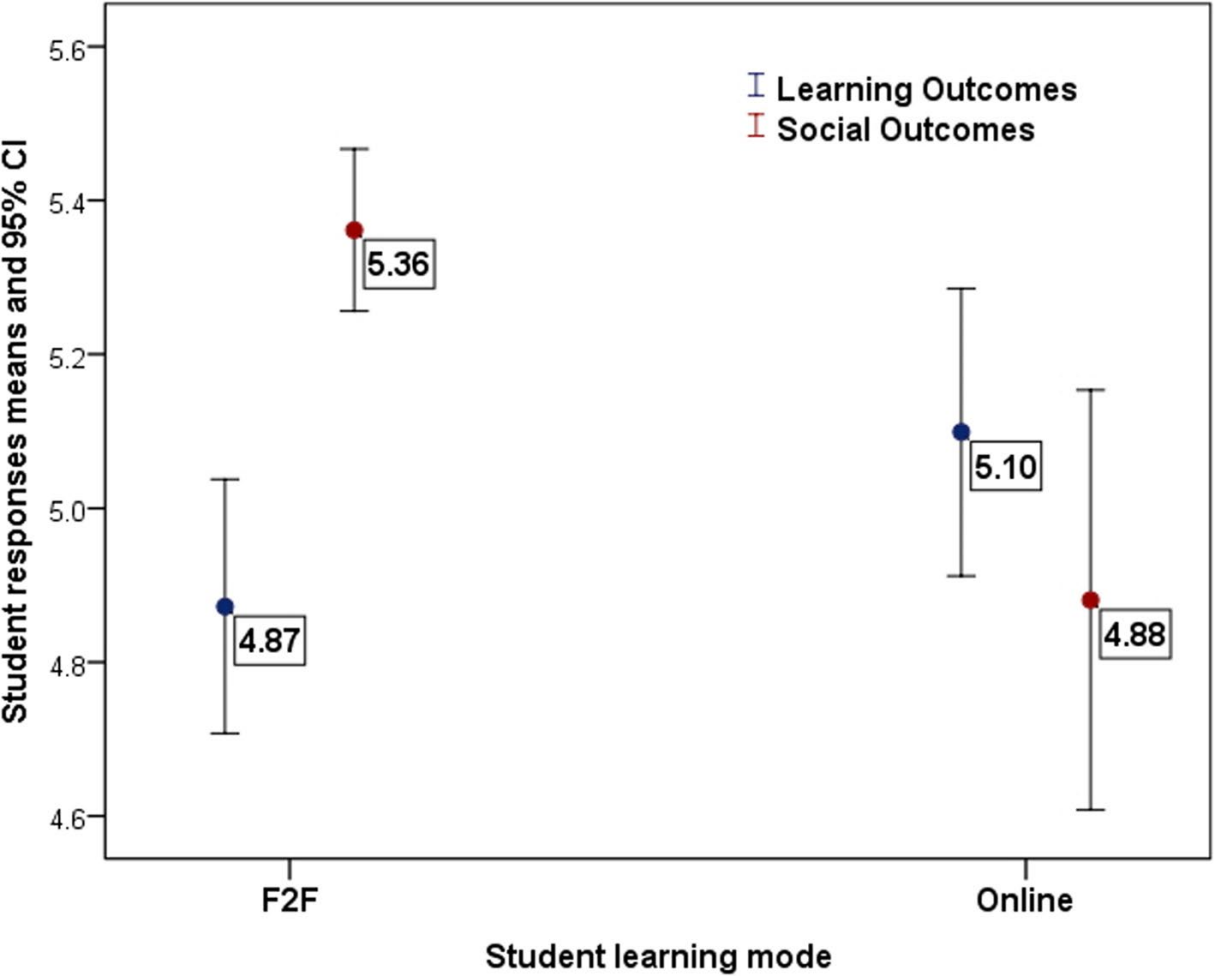
Impact of learning mode (online/F2F). Note: Z and Y-axes do not intersect at zero

The impact of format on learning and social outcomes is also shown in Fig. 1, which graphically shows the better social outcomes in students who had face to face scenario group teaching, with no significant difference between the groups in terms of their perceived learning outcomes.

When we used linear regression to examine the impact of both mode of learning and social outcomes on the learning outcomes, there was a high degree of reliability (as shown by the Pattern Matrix in Table 1). Linear regression revealed that the social outcomes have almost the double the impact on learning outcomes than mode of learning i.e.. f2f or online (Table 3, β = .45 and β = .23 respectively).

**Table 3:** Linear regression analysis of impact of mode of learning and social outcomes on perceived learning outcomes

		Coefficients^**a**^						
Model		Unstandardized Coefficients		Standardized Coefficients	t	Sig.	95.0% Confidence Interval for B	
		B	Std. Error	Beta			Lower Bound	Upper Bound
1	(Constant)	2.20	.39		5.60	.00	1.43	2.98
	Social outcomes	.50	.07	**.45**	6.90	.00	.36	.64
	Mode of Learning	.476	.13	**.23**	3.58	.00	.21	.72

^a^Dependent Variable: Learning Outcomes; R2 = 0.199

### Summative assessment of knowledge gains

No differences were found in relation to performance at end of course exams between offshore (international students), onshore international students and domestic students in 2021. There was also no difference when compared with historical cohorts (2020 was taught entirely online and 2019 was taught entirely face to face).

### Impact on wellbeing

**Fig. 2:**
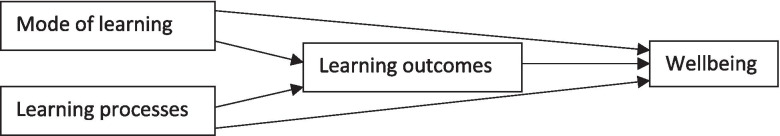
Direct and indirect impact of factors on wellbeing

In order to assess the impact of these factors (learning outcomes, social outcomes and mode of learning) on student wellbeing, we looked at both the direct and indirect impact of these variables using path analysis is illustrated in Fig. 2 [9].

A positive rating of the learning outcomes had the most significant positive correlation with ratings of wellbeing. Importantly, mode of learning did not have any direct impact on student wellbeing (Table 4). The impact of the mode of learning on wellbeing was insignificant.

**Table 4:** Effect of variables (factors) on Wellbeing (Path Analysis)

Direct, Indirect and Total Effects								
				95% C.I. (a)				
Type	Effect	Estimate	SE	Lo	Hi	β	z	p
Indirect	Mode of Learning - > Learning Outcomes - > Enhanced Wellbeing	0.29	0.09	0.12	0.46	0.12	3.37	< .001
	Social Outcomes - > Learning Outcomes - > Enhanced Wellbeing	0.31	0.05	0.21	0.42	0.24	5.72	< .001
Component	Mode of Learning - > Learning Outcomes	0.46	0.13	0.21	0.72	0.23	3.57	< .001
	Learning Outcomes - > Enhanced Wellbeing	0.63	0.06	0.51	0.75	0.54	10.12	< .001
	Social Outcomes - > Learning Outcomes	0.50	0.07	0.36	0.64	0.45	6.93	< .001
Direct	Mode of Learning - > Enhanced Wellbeing	−0.02	0.12	− 0.25	0.22	− 0.01	− 0.16	0.87
	Social Outcomes - > Enhanced Wellbeing	0.44	0.07	0.30	0.58	0.33	6.13	< .001
Total	Mode of Learning - > Enhanced Wellbeing	0.27	0.14	−0.01	0.55	0.12	1.91	0.056
	Social Outcomes - > Enhanced Wellbeing	0.75	0.08	0.60	0.90	0.57	9.53	< .001

*Note*. Confidence intervals computed with method: Standard (Delta method)

### Qualitative results

Thematic text extracts were automatically produced by Qualtrics with inherent sentiment analysis and were similar across both groups in relation to the themes and best aspects of the SGs. This showed us that the best thing reported by the most students were the facilitators (teachers) of the groups. Other positive themes that emerged included: learning in small groups, revisiting lecture content, creation of a safe environment, interacting with peers, sharing ideas and making friends.

## Discussion

As medical educators reflect on and evaluate the rapid changes that occurred out of necessity during 2020 and 2021 [12–17], it will be important to evaluate what changes can and should remain, what the impact of various changes is on student learning and how these changes affect other parts of the student experience, such as wellbeing and engagement. This is particularly relevant to the teaching in the junior campus-based years of medical programs, where there can be many benefits of shifting educational activities to an online format. These include flexibility (geographical and time-related), scalability, interactivity, and quality control [12, 18]. Students have given us feedback that all these things are better in the online environment, if appropriate effort is put into the design of educational resources [12, 19]. Online teaching in medical programs is not new, but has been expedited during COVID-19 [20]. One very important finding in our data set is that the mode of learning (online versus f2f) did not actually have an impact on learning outcomes of students either from the student’s perception or objectively measured via the summative end-of-course examinations. This major finding has also been reported elsewhere [21, 22]. Two meta-analyses that have examined learning knowledge gains, skill gains and satisfaction of medical students have also found no difference between learning in a f2f environment and slightly better performance in the online environment for some parametres [23, 24].

Our study found that the mode of teaching was much less important than the social outcomes (engagement with peers and facilitators, making friends) on both learning outcomes and student wellbeing. Learning outcomes and knowledge gains can be maintained and optimised if appropriate work is put into educational design [24]. In fact, the opportunity (and need) to develop learning materials for online delivery of healthcare education, may have enhanced these activities by supporting instructional design and embedding opportunity for rapid feedback [25, 26]. One of the challenges in e-learning is that not all activities work well in the online environment and this needs to be taken into consideration, when designing the activities [18]. Other studies have also highlighted the fact that many teaching formats do not translate well ‘online’ and that this mode may be more suited to junior rather than senior (clinical) years of most programs [27].

Our study found that the social processes which include engagement with peers and facilitators and making friends was affected by the mode of delivery. This is not surprising, given that the small group learning such as SGs are the main learning activity during which these social processes can occur for the students. The group work arising out of SGs often require students to organise study sessions in addition to formal timetabled ones, allowing friendships to flourish and more informal communication. This is more constrained when all these activities must occur online [28].

Wellbeing was affected by both learning outcomes and social outcomes but not independently by mode of learning. The impact of social outcomes such as making friends and engaging with peers and facilitators had double the impact of the learning outcomes. It has also been shown elsewhere that the development of supportive relationships with peers and staff is very important for student wellbeing [29, 30] . Issues which impact the building of peer relationships and friendships, such as the constraints of an entirely online environment, have a negative impact on wellbeing [12, 18, 29]. Other studies have also shown that stress relating to academic performance has also been shown to have a negative impact on mental health in medical students and many medical programs are now being proactive in efforts to address this [29, 31].

Our qualitative data also tells us that in both environments, the impact of the facilitator on the student experience is very important. In the online environment, the facilitator has an even greater role in keeping students engaged and focused [32]. It is also likely that facilitators benefit from specific training on how to best teach in this environment, as there are issues to manage that do not arise in the f2f environment, such as the online etiquette, building physically-distanced community for collaborative learning, and the use of specific online tools [14, 33, 34].

### Limitations of this study

It was beyond the scope of this study to assess the impact of mode of delivery on the development of a range of individual skills and competencies taught across courses in medical programs and gradually developed by students, that may be more affected by mode of learning such as teamwork, communication, professionalism, and cultural safety. As mentioned in the meta-analysis by Pei [21] there are many items and factors that could be used in the analysis of online learning, in this study we have only looked at a limited number of items. This study was undertaken in first year students and so the findings relate to teaching in junior years in medical programs. They may not be generalisable to learning in the senior years or in the clinical environment. It is also important to acknowledge that this study was undertaken during the ‘COVID pandemic’ which may have impacted on the student responses in some ways which would be different in a non-pandemic time.

## Conclusion

Ultimately the findings of our study support the idea that educational design and delivery has a greater impact, than the mode of delivery on both learning outcomes and processes (such as motivation, satisfaction, self-perceived knowledge gains) and social processes (engagement with peers and facilitators). Social processes are more impacted than the learning ones by mode and this can have an impact on student wellbeing. This study highlights the need for educators and students to put additional thought into how to address these aspects in the online learning environment. This data emphasises the need for high quality educational design and iterative improvement whatever format is used [24, 35] as well as support and development of highly competent educators who are environment ambidextrous, so that student engagement is optimised [36]. Putting energy into the development of contemporary educational resources and the development of our facilitators also builds resilience into our programs, our staff and our students, improves equity and will set medical programs up well against future disruptions.

## Supplementary Information

**Additional file 1.**

**Additional file 2.**

## Data Availability

The datasets used and/or analysed during the current study are available from the corresponding author on reasonable request.

## Abbreviations

f2f: Face to face
SG: Scenario group
COVID-19: SARS-CoV-2 virus
