# Supplementary material for: It’s what you do, not the way you do it – online versus face-to-face small group teaching in first year medical school
Source: BMC Med Educ. 2021 Oct 26;21:541. doi: 10.1186/s12909-021-02981-5 (PMC8546782; doi:10.1186/s12909-021-02981-5)
Supplement: Supplementary file 1 — Additional file 1. [file 12909_2021_2981_MOESM1_ESM.docx]

**Appendix A – Questions included in the survey**

1. The format for the scenario group (SG) I was in was:
   1. Face to face
   2. Online

Responses on a Likert scale of 1-6

1. I was satisfied with the scenario group (SG) sessions
2. I enjoyed the learning format (online or face to face) for the SG sessions
3. The SG sessions enhanced my motivation to learn in this course
4. The SG sessions provided activities that enhanced my learning
5. The SG sessions were useful for increasing my knowledge
6. It was easy to contribute to the group during the SG sessions
7. I found it easy to engage with my facilitator in the SG sessions
8. I found it easy to make connections with other students that enhanced my learning
9. I found it easy to make friends with other students in my SG
10. I felt that the SG sessions enhanced my wellbeing

Open response:

1. Please comment on the best features of the SG sessions:
