# Supplementary material for: It’s what you do, not the way you do it – online versus face-to-face small group teaching in first year medical school
Source: BMC Med Educ. 2021 Oct 26;21:541. doi: 10.1186/s12909-021-02981-5 (PMC8546782; doi:10.1186/s12909-021-02981-5)
Supplement: Supplementary file 2 — Additional file 2. [file 12909_2021_2981_MOESM2_ESM.docx]

Appendix B. Likert scale question results

1. F2f cohort

| # | Field | Minimum | Maximum | Mean | Std Deviation | Variance | Count |
| --- | --- | --- | --- | --- | --- | --- | --- |
| 1 | I was satisfied with the SG sessions | 1.00 | 6.00 | 5.12 | 0.98 | 0.97 | 143 |
| 2 | I enjoyed the learning format (online or face to face) for the SG sessions | 2.00 | 6.00 | 5.48 | 0.78 | 0.61 | 143 |
| 3 | The SG sessions enhanced my motivation to learn in this course | 1.00 | 6.00 | 4.82 | 1.17 | 1.37 | 143 |
| 4 | The SG sessions provided activities that enhanced my learning | 1.00 | 6.00 | 4.83 | 1.15 | 1.32 | 143 |
| 5 | The SG sessions were useful for increasing my knowledge | 1.00 | 6.00 | 4.74 | 1.15 | 1.33 | 143 |
| 6 | It was easy to contribute to the group during the SG sessions | 2.00 | 6.00 | 5.19 | 0.95 | 0.90 | 143 |
| 7 | I found it easy to engage with my facilitator in the SG sessions | 2.00 | 6.00 | 5.47 | 0.76 | 0.57 | 143 |
| 8 | I found it easy to make connections with other students that enhanced my learning | 3.00 | 6.00 | 5.37 | 0.83 | 0.69 | 143 |
| 9 | I found it easy to make friends with other students in my SG | 3.00 | 6.00 | 5.43 | 0.78 | 0.61 | 143 |
| 10 | I felt that the SG sessions enhanced my wellbeing | 1.00 | 6.00 | 4.96 | 1.09 | 1.19 | 143 |

2. Online cohort

| # | Field | Minimum | Maximum | Mean | Std Deviation | Variance | Count |
| --- | --- | --- | --- | --- | --- | --- | --- |
| 1 | I was satisfied with the SG sessions | 2.00 | 6.00 | 5.20 | 0.91 | 0.84 | 65 |
| 2 | I enjoyed the learning format (online or face to face) for the SG sessions | 2.00 | 6.00 | 4.94 | 1.13 | 1.28 | 65 |
| 3 | The SG sessions enhanced my motivation to learn in this course | 2.00 | 6.00 | 4.97 | 0.99 | 0.98 | 65 |
| 4 | The SG sessions provided activities that enhanced my learning | 3.00 | 6.00 | 5.19 | 0.81 | 0.66 | 63 |
| 5 | The SG sessions were useful for increasing my knowledge | 4.00 | 6.00 | 5.08 | 0.81 | 0.66 | 65 |
| 6 | It was easy to contribute to the group during the SG sessions | 2.00 | 6.00 | 5.06 | 1.04 | 1.07 | 65 |
| 7 | I found it easy to engage with my facilitator in the SG sessions | 2.00 | 6.00 | 5.26 | 1.10 | 1.21 | 65 |
| 8 | I found it easy to make connections with other students that enhanced my learning | 1.00 | 6.00 | 4.63 | 1.33 | 1.77 | 65 |
| 9 | I found it easy to make friends with other students in my SG | 1.00 | 6.00 | 4.57 | 1.42 | 2.03 | 65 |
| 10 | I felt that the SG sessions enhanced my wellbeing | 2.00 | 6.00 | 4.86 | 1.11 | 1.23 | 65 |
